# Bronchobiliary fistula after radiofrequency ablation for hepatocellular carcinoma successfully treated by double drainage

**DOI:** 10.1002/rcr2.376

**Published:** 2018-10-25

**Authors:** Yi Chen Chang, Yu Min Lin

**Affiliations:** ^1^ Department of Surgery Shin Kong Wu Ho‐Su Memorial Hospital Taipei Taiwan; ^2^ Department of Hepatogastroenterology Shin Kong Wu Ho‐Su Memorial Hospital Taipei Taiwan

**Keywords:** Bronchobilary fistula, endoscopic retrograde biliary drainage, hepatocellular carcinoma, percutaneous transhepatic drainage, radiofrequency ablation

## Abstract

For hepatocellular carcinomas, radiofrequency ablation is extensively used to alleviate primary and metastatic hepatic tumours. Common complications of this procedure include bleeding, infection, and hollow organ perforation. We present the case of a patient with hepatoma who underwent radiofrequency ablation. He had intractable cough with yellowish sputum, particularly while lying down, three weeks after treatment. Chest computed tomography demonstrated a right middle lobe consolidation with pleural effusion and right subphrenic fluid collection. Thoracoscopic decortication was performed under the diagnosis of empyema. The attending anaesthesiologist noted bile‐like fluid aspirated from the endotracheal tube. Therefore, we suspected bronchobiliary fistula. Percutaneous transhepatic drainage of the subphrenic fluid and simultaneous cholangiography confirmed bronchobiliary fistula. The patient was successfully treated using percutaneous drainage combined with endoscopic retrograde biliary drainage. An imaging finding of subphrenic fluid collection with right lower lung consolidation after radiofrequency ablation for hepatic tumours should raise the suspicion of bronchobiliary fistula.

## Introduction

Bronchobiliary fistula is an abnormal connection between the hepatobiliary system and the respiratory system. In general, the leading causes of bronchobiliary fistula include local infection, biliary tract obstruction, neoplasm, trauma, and surgery. The most common and typical symptom of the condition is biliptysis. Because this disorder is rare, we were not sufficiently able to make an appropriate diagnosis and, thus, misdiagnosed the patient as having pneumonia with empyema in this case study. The standard diagnostic method is percutaneous or endoscopic retrograde cholangiography, which demonstrates an abnormal tract between the liver and the lungs.

## Case Report

**Figure 1:**
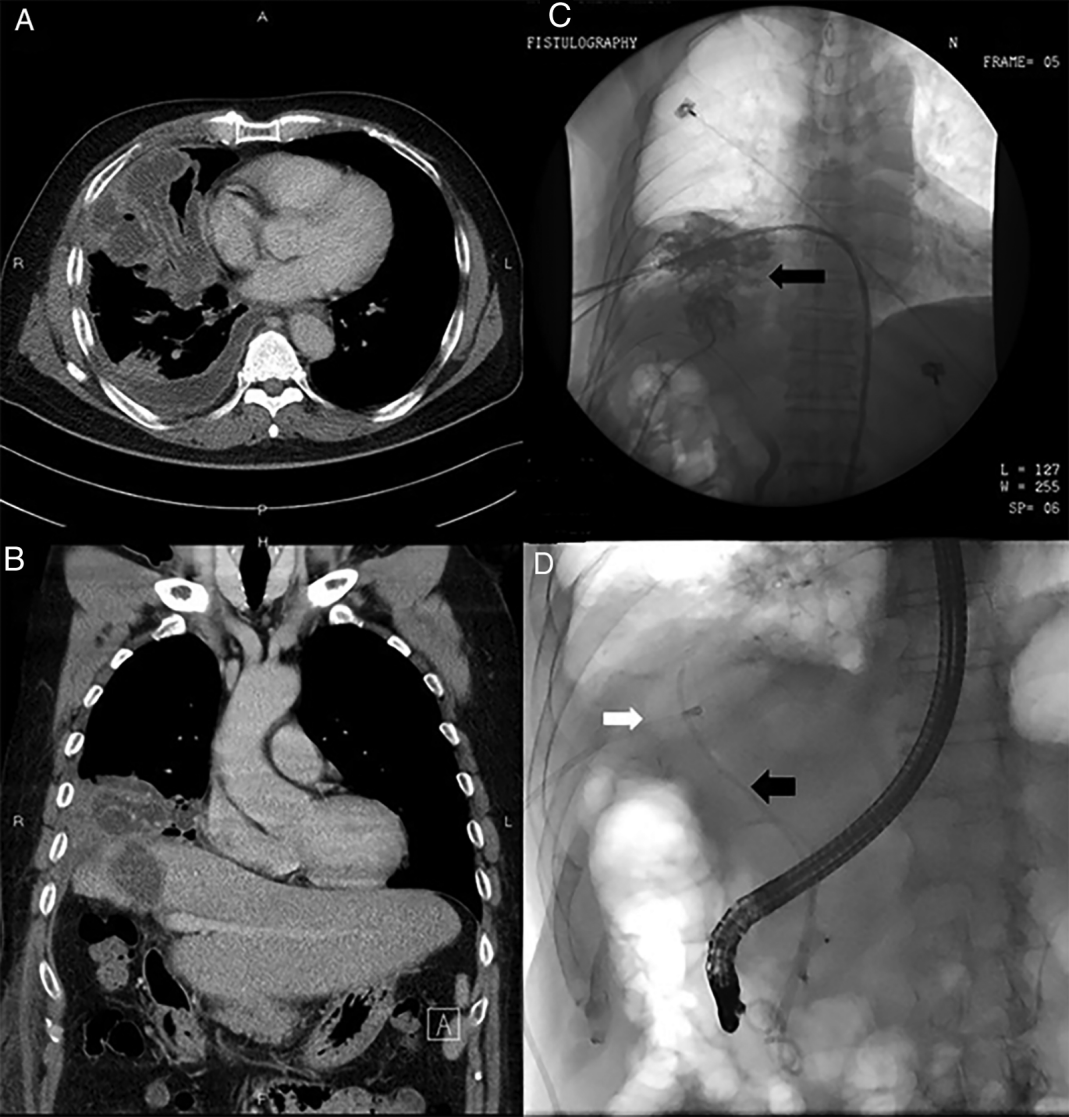
(A) Computed tomography demonstrated right middle lobe consolidation with pleural effusion. (B) Right subphrenic fluid collection. (C) Percutaneous cholangiography indicated the bronchobiliary fistula. (D) Percutaneous drainage (white arrow) combined with endoscopic retrograde biliary drainage (black arrow).

We present the case of a 61‐year‐old man with chronic hepatitis B with liver cirrhosis (Child A class) and hepatocellular carcinoma. He underwent right‐lobe hepatectomy in 2009. However, the cancer recurred, and he underwent transarterial chemoembolization seven times. In February 2017, he underwent radiofrequency ablation for the recurrent hepatoma. Three weeks after the treatment, he presented with the concern about intractable cough with yellowish sputum. Chest film examination indicated right lower lung consolidation; moreover, computed tomography demonstrated right middle lobe consolidation with pleural effusion and right subphrenic fluid collection (Fig. 1A and B). The laboratory findings also show abnormality (CRP 28.5 mg/dL, ALP 121 U/L, r‐GT 111 U/L, Bil T/D 3.18/1.79 mg/dL, AST 16 U/L, ALT 17 U/L). Right‐side subphrenic abscess with empyema thoracis was the tentative diagnosis, and surgical drainage of the observed empyema was performed. During the perioperative period, the attending anaesthesiologist noted an aspiration of yellowish clear fluid from the endotracheal tube, and bronchobiliary fistula was suspected. Percutaneous echo‐guided transhepatic cholangiography demonstrated an abnormal tract from the liver to the lung. Thus, this confirmed the diagnosis of bronchobiliary fistula (Fig. 1C).

Surgical intervention was not considered because the patient’s general condition was not adequate for surgery. We decided to perform percutaneous drainage. The patient’s biliptysis was considerably alleviated after the procedure. However, the amount of fluid drained did not decrease in 2 weeks, and we observed that occluding the drainage tube would lead to biliptysis recurrence. We decided to perform endoscopic retrograde biliary drainage combined with percutaneous drainage (Fig. 1D) because we assumed that a biliary tract stricture might occur after radiofrequency ablation and lead to inadequate bile drainage. The endoscopic retrograde biliary drainage tube was removed 13 days later, and the observed fistula healed at clinical presentation. Follow‐up computed tomography conducted five months after the episode demonstrated satisfactory recovery of the liver and lung parenchyma.

## Discussion

Bronchobiliary fistula is a rare disorder involving an abnormal connection between the hepatobiliary tract system and the respiratory system. In general, the leading causes of bronchobiliary fistula are local infection, biliary tract obstruction, neoplasm, trauma, and surgery. Currently, primary or metastatic neoplasms are the most common precursors of bronchobiliary fistula [1].

Biliptysis is the most typical symptom of bronchobiliary fistula. However, if a patient presents with scanty bile‐tinged sputum rather than a copious amount of bile, this symptom would be neglected. An imaging study demonstrated that lung consolidation caused by bile irritation may be misdiagnosed as pneumonia. A biochemical study of sputum is non‐invasive and can be helpful in diagnosis processes. The standard diagnostic method is percutaneous transhepatic cholangiography or endoscopic retrograde cholangiography 1, 2, which can demonstrate abnormal connections between the hepatobiliary tract and the respiratory system. The catheter could also be maintained as a drainage tube. Technological advancements have enabled the development of magnetic resonance cholangiography (MRC), a non‐invasive approach that can confirm a diagnosis. However, the most crucial aspect of a diagnostic approach is to indicate the possibility of bronchobiliary fistula.

Radiofrequency ablation is a minimally invasive and safe procedure that is extensively used for alleviating hepatic tumours. Only a few radiofrequency ablation‐induced cases of bronchobiliary fistula after treatment for hepatocellular carcinoma have been reported (Table 1) 3, 4, 5, 6. The possible causes have been indicated to be collateral thermal injury to the diaphragm, direct tumour invasion to the diaphragm, local infection after ablation, and biliary stricture after ablation. For our patient, the tumour did not invade the diaphragm, and no subphrenic abscess was observed. We attributed the detected fistula to collateral thermal damage. The biliary tract stricture observed after ablation also played a role in the development of the observed fistula.

**Table 1:** Literature review describing bronchobiliary fistula induced by radiofrequency ablation for hepatocellular carcinoma.

Author	Location of tumour	Size of tumour	Number of ablation	Power of RFA	Onset of BBF after RFA	Treatment modality	Reference
Yoon et al.	Dome	NA	2	200 W; 12 min	2 months	PD	3
Kim et al.	Dome	3.5 cm	NA	NA	48 days	Surgery	4
Dai et al.	Right posterior lobe	5.5 cm	NA	NA	20 days	Expired	5
Zeng et al.	Segment VIII	4.4 cm	8	200 W; 12 min	17 months	PD failed; surgery	6

BBF: bronchobiliary fistula, NA: not available, PD: percutaneous drainage, RFA: radiofrequency ablation.

Treatment options for bronchobiliary fistula include surgical and non‐surgical procedures 1, 2, 3, 7, 8. Surgical procedures include resection of the affected lung, repair of the diaphragm with viable tissue such as muscle flaps, and drainage of hepatic abscess, and are usually set as the final step. Percutaneous drainage, endoscopic retrograde biliary drainage, and endoscopic blockage of the fistula using histoacryl have been reported to be successful in treating bronchobiliary fistula. For the case reported in this paper, percutaneous drainage was first performed, but the procedure failed. We considered that the distal biliary tract was obstructed after the radiofrequency ablation process, leading to bile accumulation and thus engendering subphrenic biloma formation. We observed a patent tract to the respiratory system; the fluid flowed to the lung, and the tract was not sealed off. Therefore, we performed endoscopic retrograde drainage to drain the accumulated bile and percutaneous drainage to drain the accumulated biloma, thus ensuring a suitable local environment for the fistula to heal.

In conclusion, bronchobiliary fistula is a rare disorder and should be suspected if a patient presents with yellowish sputum along with a hepatobiliary disease. Diagnosis could be made using traditional cholangiography or MRC. Percutaneous drainage and endoscopic retrograde biliary drainage may successfully treat the fistula.

### Disclosure Statement

Appropriate written informed consent was obtained for publication of this case report and accompanying images.
